# Altered bone marrow lymphopoiesis and interleukin-6-dependent inhibition of thymocyte differentiation contribute to thymic atrophy during *Trypanosoma cruzi infection*

**DOI:** 10.18632/oncotarget.14886

**Published:** 2017-01-28

**Authors:** Sofía Carbajosa, Susana Gea, Carlos Chillón-arinas, Cristina Poveda, María del Carmen Maza, Manuel Fresno, Núria Gironès

**Affiliations:** ^1^ Centro de Biología Molecular Severo Ochoa, Consejo Superior de Investigaciones Científicas, Universidad Autónoma de Madrid, Cantoblanco, Madrid, Spain; ^2^ Departamento de Bioquímica Clínica, Centro de Investigaciones en Bioquímica Clínica e Inmunología, Facultad de Ciencias Químicas, Universidad Nacional de Córdoba, Córdoba, Argentina; ^3^ Instituto Sanitario de Investigación Princesa, Madrid, Spain

**Keywords:** thymic atrophy, interleukin-6, glucocorticoids, Trypanosoma cruzi infection, Chagas disease

## Abstract

Thymic atrophy occurs during infection being associated with apoptosis of double positive (DP) and premature exit of DP and double negative (DN) thymocytes. We observed for the first time that a significant bone marrow aplasia and a decrease in common lymphoid progenitors (CLPs) preceded thymic alterations in mice infected with *Trypanosoma cruzi*. In addition, depletion of the DN2 stage was previous to the DN1, indicating an alteration in the differentiation from DN1 to DN2 thymocytes. Interestingly, infected mice deficient in IL-6 expression showed higher numbers of DP and CD4^+^ thymocytes than wild type infected mice, while presenting similar percentages of DN1 thymocytes. Moreover, the drop in late differentiation stages of DN thymocytes was partially abrogated in comparison with wild type littermates. Thus, our results suggest that thymic atrophy involves a drop in CLPs production in bone marrow and IL-6-dependent and independent mechanisms that inhibits the differentiation of DN thymocytes.

## INTRODUCTION

Thymic involution and decreased thymopoiesis are highly related simultaneous events. During this process the size and function of the thymus is drastically reduced (up to 95%) which can be triggered by several processes such as aging [1], pregnancy [2] or stress [3]. Thymic involution is observed also during infection, existing two main hypothesis about its advantages and disadvantages, as the prevention of tolerance against pathogen's antigens [4], and as a cause of immunosuppression [5], respectively.

The maturing T cells (thymocytes) can be classified (according to CD4 and CD8 markers expression) in the following major stages: about 5% are double negative (DN) thymocytes, 80% are double positive (DP) thymocytes, 10% are CD4 single positive (SP) and 5% are CD8 SP [6]. T cell development in the thymus progresses from DN to DP and finally to CD4 or CD8 SP [7]. In addition, DN thymocytes can be subdivided in 4 stages (from DN1 to DN4) characterized by differential expression of CD25 and CD44 markers.

During the acute phase of *Trypanosoma cruzi* infection, the causal agent of Chagas disease, the thymus undergoes an involution that has been associated to a drastic reduction of DP thymocytes in the thymus [8]. Several mechanisms, which are not mutually exclusive, can alter normal thymic development and explain this involution: (I) the mobilization of DP thymocytes to peripheral organs, as detected by observing DP T cells in secondary lymphoid organs [9, 10], (II) apoptotic death of DP thymocytes [11] that has been linked to various causes such as trans-sialidase activity of the parasite [12] extracellular ATP [13] or dysregulation of positive selection due to excessive production of glucocorticoids [14] and (III) premature exit of DN cells from the thymus [15]. Moreover, bone marrow B cell maturation is also altered during *T. cruzi* infection [16].

On the other hand, we previously observed DP depletion in mice susceptible and non-susceptible to *T. cruzi* infection, being the susceptibility associated with high levels of IL-6 in plasma [17]. Interestingly, IL-6 has also been involved in some cases of thymus atrophy, as thymic depletion due to age [1] or fetal thymic atrophy caused by LPS treatment [18], indicating a possible link between IL-6 and thymic alterations.

IL-6 is mostly produced in the thymus by mature I-A and Mac-1 positive accessory cells [19], and by thymic epithelial cells (TECs) [20]. IL-6 has been described to act both as an anti- and pro-inflammatory cytokine, depending on its receptor binding. After IL-6 binding to the IL-6α receptor (IL-6Rα) it subsequently binds to the IL-6Rβ (gp130) receptor, and there is increased proliferation and inhibition of apoptosis, a process known as classic-signaling [21]. On the contrary, sometimes a soluble form of the IL-6R is shed from the membrane and binds to IL-6 which can interact directly on cells expressing membrane gp130, but not IL-6R, activating a signaling pathway known as trans-signaling [21].The later exerts a pro-inflammatory role, activating the immune system at several levels as recruitment of mononuclear cells through CCL2, inhibition of T-cell apoptosis and inhibition of regulatory T cell differentiation [21].

In view of the above, we studied whether (I) a decrease in thymocyte bone marrow precursors could influence the levels of thymic populations and (II) that IL-6 induced by infection alter thymocyte differentiation contributing to thymic atrophy. We found a significant decrease in bone marrow CLPs and DN1 thymocyte stage, both independent of IL-6, prior to any other alteration in the thymus. Notably, we also describe for the first time that *T. cruzi* infection altered DN1 to DN2 stage transition leading to deficient thymocyte differentiation. This effect was dependent on IL-6, since it was partially abrogated in mice deficient in IL-6 expression. Moreover, IL-6 deficient mice showed lower thymic depletion than wild type animals after infection.

## RESULTS

### Bone marrow aplasia and CLPs in *T. cruzi* infected mice

**Figure 1:**
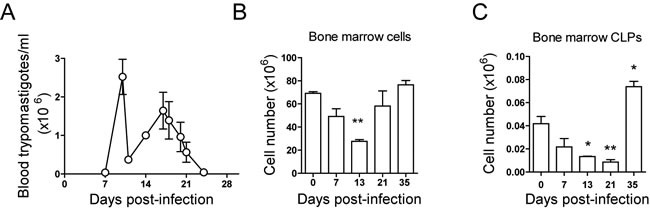
Bone marrow cells and CLPs during *T. cruzi* infection BALB/c mice were infected with *T. cruzi*, bone marrow cells and thymocytes were obtained at different days post-infection and counted under microscopical examination in Neubauer chambers. **A**. Number of trypomastigotes per ml of blood during *T. cruzi* infection. **B**. Total number of bone marrow cells. **C**. Total number of CLPs was determined by analyzing Lin^neg^ cells, Sca-1^neg^ c-Kit^med^. A representative experiment out of two independent experiments is shown. One way ANOVA tests were performed to determine the significance of the results (*n* = 3; * *p* < 0.05; **p < 0.01; ***p < 0.001).

Mice infected with *T. cruzi* showed high levels of parasitemia between days 7 and 21 after infection (Figure 1A). We first examined the status of the major and primary hematopoietic organ, the bone marrow, during the acute phase of infection. Bone marrow total cell numbers were significantly decreased at day 13 post-infection and recovered the basal levels, by day 21 post-infection (Figure 1B). Lin^neg^ Sca-1^neg^ c-Kit^med^ corresponding to common lymphocyte precursors (CLPs) analysis showed that cell percentages in bone marrow of infected mice did not significantly change respect non-infected mice (Supplemental Figure 1A). However, accordingly with total bone marrow cell numbers, we found a significant decrease in CLPs numbers at 13 and 21 d.p.i. (Figure 1C) that reached levels above the basal by day 35 post-infection. These results suggest that bone marrow aplasia and CLPs low numbers can contribute to thymic atrophy.

### Dynamics of thymocyte stages in *T. cruzi* infected mice

**Figure 2:**
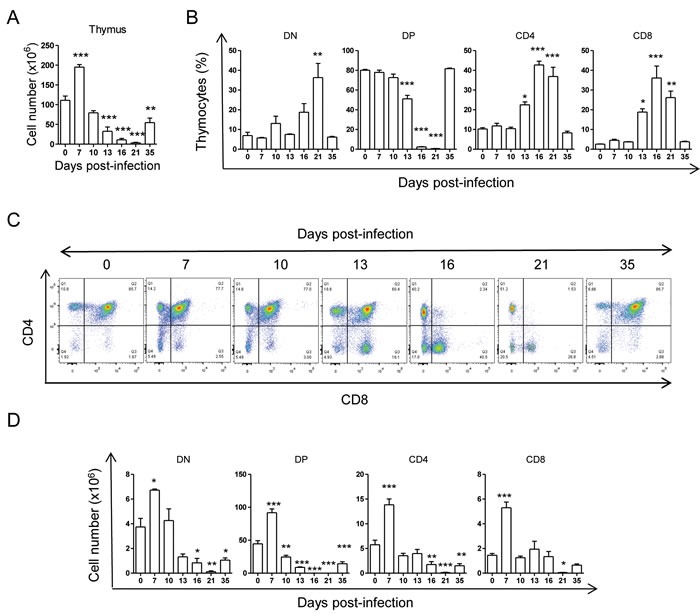
*Thymic DN, DP and SP stages during T.* cruzi infection BALB/c mice were infected with *T. cruzi*, thymus were obtained at different days post-infection and analyzed by Flow Cytometry as described in material and methods. **A**. Total thymocyte numbers. **B**. Dot plot analysis of thymic cells from representative mice based on CD4 and CD8 markers showing DN, DP, CD4+ and CD8+stage percentages at different d.p.i. **C**. Graphs representing mean ± SEM of the percent of DN, DP, CD4+ and CD8+ thymocyte percentages at different d.p.i.. **D**. Graphs representing the total number of each DN, DP, CD4+ and CD8+ thymocyte stages at different d.p.i. A representative experiment out of six independent experiments is shown. One way ANOVA tests were performed to determine the significance of the results (*n* = 3; * *p* < 0.05; **p < 0.01; ***p < 0.001)..

We next analyzed the cellularity in the thymus. Thymocyte numbers significantly increased by day 7 post-infection but dropped significantly at 13, 16, 21 and 35 days post-infection (d.p.i.) (Figure 2A). As previously observed [17, 22], analysis of thymic differentiation of DN, DP and SPs thymocytes regarding CD4 and CD8 markers during the acute infection, showed a huge decrease in the percentages (Figure 2B and 2C) and total numbers (Figure 2D) of DP thymocytes, starting at 13 d.p.i. and partially recovering later in infection. As for total thymocytes (Figure 2A) at day 7 post-infection the number of DP and SP thymocytes showed an increment in total cell numbers (Figure 2D). Notably, total DN thymocyte numbers, but not percentages, significantly decreased upon infection, being this discrepancy due to the huge decrease in the major DP populations (Figure 2B, 2C and 2D). On the other hand, the relative percentages of both CD4^+^ and CD8^+^ SPs thymocytes were increased at 13, 16 and 21 d.p.i. also likely due to the severe depletion of DP cells (Figure 2B and 2C), but total numbers dropped dramatically at 21 and 35 d.p.i. (Figure 2D). Thymocyte populations tend to recover at 35 d.p.i., likely indicating a transition to the chronic phase of infection.

### Alteration of DN thymocyte development in *T. cruzi* infected mice

**Figure 3:**
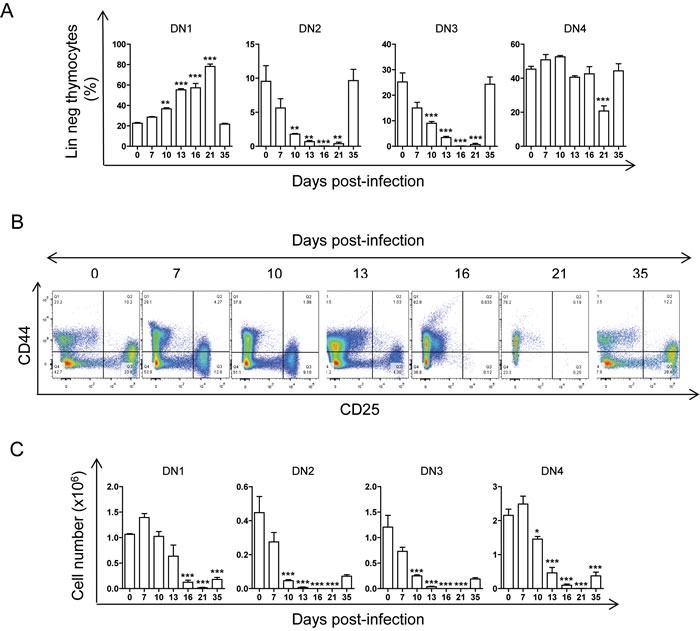
Thymic DN1-DN4 stages during *T. cruzi* infection BALB/c mice were infected with *T. cruzi*, thymus were obtained at different days post-infection and analyzed by Flow Cytometry as described in material and methods. **A**. Dot plot analysis of thymic cells from representative mice based on CD25 and CD44 markers showing DN1, DN2, DN3 and DN4 thymocyte stage percentages at different d.p.i. **B**. Graphs representing mean ± SEM of the percent of DN1-4 thymocyte stages at different d.p.i. **C**. Graphs representing the total number of each DN thymocyte stage at different d.p.i. A representative experiment of two independent experiments is shown. One way ANOVA tests were performed to determine the significance of the results, (*n* = 3; * *p* < 0.05; **p < 0.01; ***p < 0.001).

We next analysed the dynamics of double negative stages with the CD25 and the CD44 markers on Lin^neg^ thymocytes to track the stages of DN thymocytes (DN1, DN2, DN3 and DN4) during infection using the gating strategy shown in supplemental Figure 1B. Interestingly, we observed that the percentages of DN2, DN3 and DN4, but not DN1, thymocytes decreased during acute *T. cruzi* infection until day 21 (Figure 3A and 3B), suggesting an alteration at this point in thymocyte differentiation caused by acute infection. However, a severe drop in total cell numbers of DN stages including DN1 was observed. Indeed, DN2, DN3, and DN4 cell numbers were reduced from day 10 post-infection and by more than 90% at 16 and 21 d.p.i. In contrast, the reduction of DN1 cell numbers was proportionally lower and occurred later (16 and 21 d.p.i.) than DN2-DN4 reduction (Figure 3C).

### IL-6 mediates a blockade in DN thymocyte differentiation in *T. cruzi* infected mice

**Figure 4:**
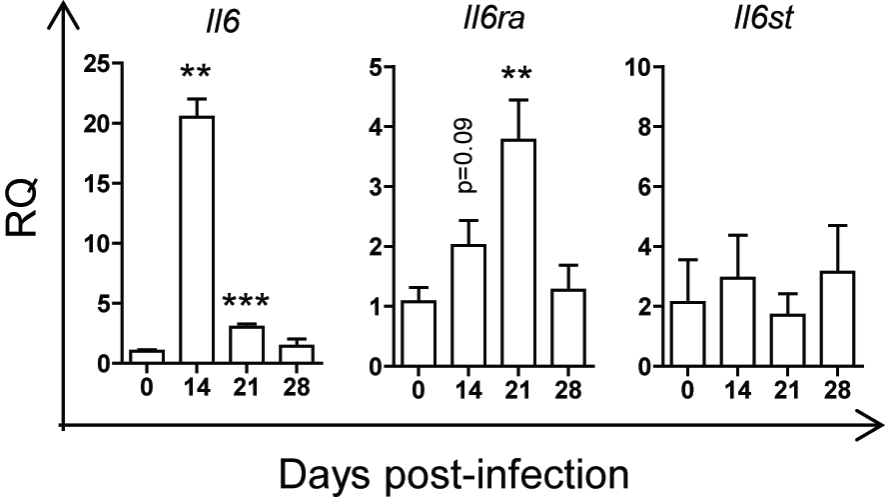
mRNA gene expression in thymus of mice infected with *T. cruzi* BALB/c mice were infected with *T. cruzi*, thymus were obtained at different days post-infection and analyzed by RTqPCR as described in materials and methods. Gene expression of *Il6*, *Il6ra* (IL-6 receptor α), *Il6st* (IL-6 receptor β or gp130), was analyzed and relative quantities (RQ) were normalized respect non-infected mice. Graphs represent mean ± SEM values. A representative experiment out of two independent experiments is shown. Student *t*-tests were performed to determine the significance of the results in infected *versus* non-infected mice. (*n* = 3; * *p* < 0.05; **p < 0.01; ***p < 0.001).

We previously observed that *T. cruzi* infection caused the increase in the levels of *Il6* mRNA expression in heart tissue and IL-6 in plasma, associated with higher pathology [17]. To address the role of IL-6 in thymocyte differentiation during infection, we first analysed the mRNA gene expression of IL-6 and its receptors in the thymus. The results showed a significant increase of IL-6 (*Il6*) at 14 and 21 d.p.i. and IL-6Rα (*Il6ra*), but not IL-6Rβ (*Il6st*), at 21 d.p.i. (Figure 4).

**Figure 5:**
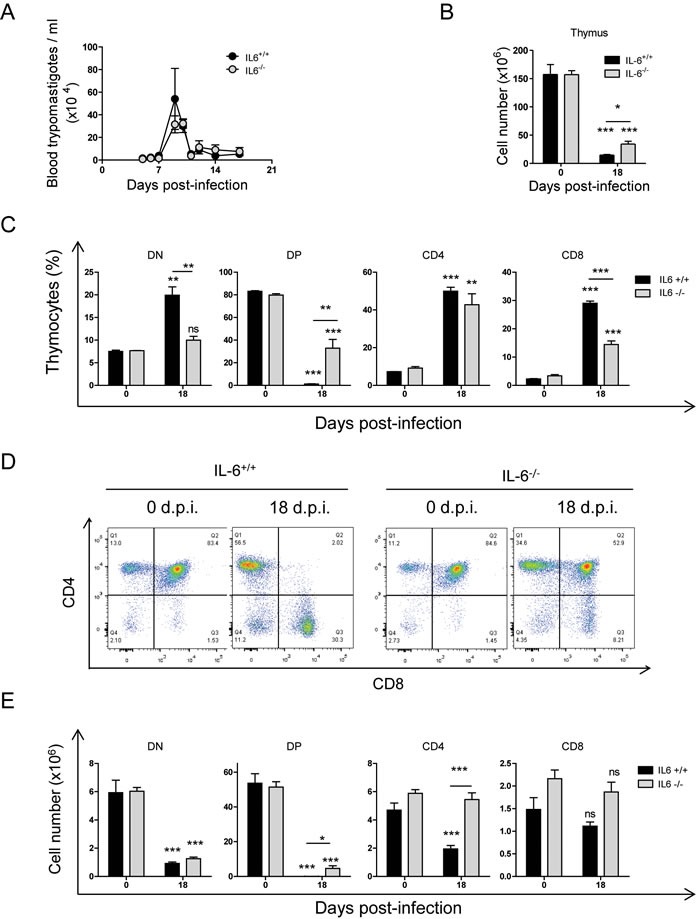
*Effect of IL-6 on thymic DN, DP and SP stages during T.* cruzi infection IL-6+/+ and IL-6−/− mice were infected with *T. cruzi*, thymus were obtained at 18 days post-infection and analyzed by Flow Cytometry as described in material and methods. **A**. Number of trypomastigotes per ml of blood during *T. cruzi* infection. **B**. Total number of thymocytes at different d.p.i. **C**. Dot plot analysis of thymic cells from representative mice based on CD4 and CD8 markers showing DN, DP, CD4+ and CD8+ thymocyte stage percentages. Graphs representing mean ± SEM of the percent of each thymocyte population. **D**. Graphs representing the total number of each DN, DP, CD4+ and CD8+thymocyte stages. A representative experiment out of two independent experiments is shown. Student *t*-tests were performed to determine the significance of the results (*n* = 3; * *p* < 0.05; **p < 0.01; ***p < 0.001).

**Figure 6:**
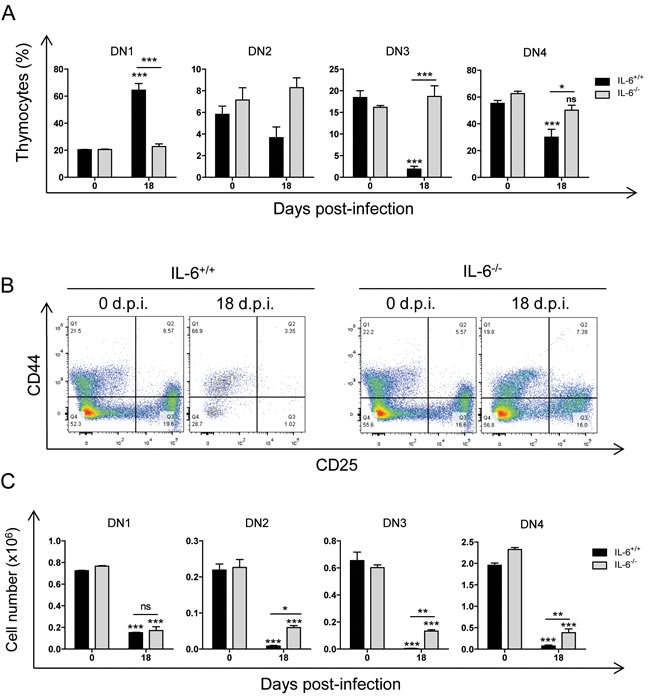
Effect of IL-6 on thymic DN1-DN4 stages during *T. cruzi* infection IL-6^+/+^ and IL-6^−/−^ mice were infected with *T. cruzi*, thymus were obtained at 18 days post-infection and analyzed by Flow Cytometry as described in material and methods. **A**. Dot plot analysis of thymic cells from representative mice based on CD25 and CD44 markers showing DN1, DN2, DN3 and DN4 stage percentages. **B**. Graphs representing mean ± SEM of the percent of DN1-4 thymocyte stages. **C**. Graphs representing the total number of each DN thymocyte stage. A representative experiment out of two independent experiments is shown. Student *t*-tests were performed to determine the significance of the results (*n* = 3; *p < 0.05; **p < 0.01; ***p < 0.001).

To better elucidate the possible involvement of IL-6 in thymic alterations caused by *T. cruzi* infection we analysed this in IL-6 deficient (IL-6^−/−^) mice. Both IL-6^+/+^ and IL-6^−/−^ infected mice showed similar levels of parasitemia around 10 d.p.i. (Figure 5A). IL-6^+/+^(C57BL/6) mice, as in BALB/c mice (Figure 2), presented a severe drop in total thymic cells upon *T. cruzi* infection (Figure 5B, black bars), as well as a huge reduction in DP and DN and increases in CD4^+^ SP total numbers (Figure 5D, black bars), indicating that there were no major differences between BALB/c and C57BL/6 strains, as we previously described [17]. Interestingly, analysis of thymocyte subpopulations (Supplemental Figure 1B) in infected IL-6^−/−^ mice showed significant differences respect to infected IL-6^+/+^mice (Figure 5 and 6, gray bars). Although a decrease in total thymocyte numbers was also observed in infected IL-6^−/−^ mice, this was significantly less pronounced than in IL-6^+/+^ mice, since more than twice the numbers of thymocytes were recovered from *T. cruzi*-infected IL-6^−/−^ mice (Figure 5B). Significant changes in the percentages of DP were detected between both strains of mice (Figure 5C and 5D). Notably, DP percentages and cell numbers were significantly higher in IL-6^−/−^ compared to IL-6^+/+^ infected mice (Figure 5D).

Interestingly, there were significantly higher DN thymocyte percentages and numbers in IL-6^−/−^ mice compared to IL-6^+/+^ mice (Figure 6A-6C). However, a significant DN total cell number reduction was observed in both IL-6^−/−^ and IL-6^+/+^ infected mice respect uninfected in all the stages analysed (Figure 5E), indicating an effect prior to DN1 that was IL-6-independent.

### Apoptosis of thymocytes during *T. cruzi* infection

**Figure 7:**
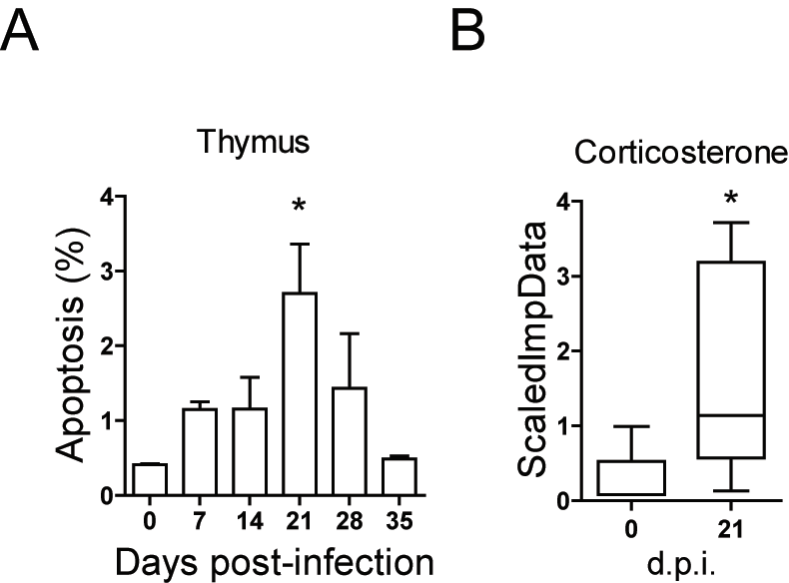
Apoptosis in thymocytes after *T. cruzi* infection BALB/c mice were infected and thymocytes and plasma were isolated as described in materials and methods. **A**. Apoptosis of thymocytes at 0, 7, 14, 21, 28 and 35 days post-infection was determined by flow cytometry by TUNEL assay as described in materials and methods. A representative experiment out of two independent experiments is shown. Student *t*-tests were performed to determine the significance of the results (*n* = 3; *p < 0.05). **B**. Corticosterone in plasma was determined in one experiment by Metabolon Inc. at 0 and 21 d.p.i. Student *t*-tests were performed to determine the significance of the results (*n* = 6; *p < 0.05).

Apoptosis is one of the mechanisms responsible for thymic atrophy, thus we performed TUNEL assays on thymic sections from infected BALB/c mice. The results showed a significant increase of apoptotic thymocytes at 21 d.p.i (Figure 7A). In addition, the levels of corticosterone, which induce thymocyte apoptosis [23], were significantly increased at 21 d.p.i. (Figure 7B). Thus, the results indicate that thymocyte apoptosis is likely increased by circulating glucocorticoids (corticosterone).

## DISCUSSION

Our results show for the first time that the decreased numbers of lymphocyte bone marrow precursors determines in a great extent the drop in the number of DN thymic populations after *T. cruzi* infection. In addition, studies in IL-6^−/−^ mice, revealed the involvement of IL-6 in decreasing the number of DN2 thymocytes after infection. Hence, both phenomena likely contribute to the drop of DP and SP thymocytes and thymic atrophy observed during infection.

Thymic atrophy during *T. cruzi* infection has been associated with premature DP and DN egress from the thymus to peripheral lymphoid organs [10, 12, 23], as well as apoptosis of DP thymocytes [24]. However, the effect of infection on bone marrow thymocyte precursors and DN thymocyte stages had never been reported before.

The bone marrow aplasia and decrease in bone marrow CLP numbers observed were temporarily coincident with the thymic atrophy.

These lymphopoietic alterations occurred prior to the increase in the apoptosis of DP thymocytes. Furthermore, infection also altered earliest steps of thymocyte differentiation in the thymus as the transition from DN1 to DN2 stages. This was suggested by a reduction in the percentages of DN2, DN3 and DN4, but not DN1, thymocytes and by an earlier and stronger reduction after infection of DN2, DN3 and DN4 respect to DN1 total cell numbers.

We also observed thymocyte apoptosis after *T. cruzi* infection as described [11], but with maximal activity after the alteration in transition from DN1 to DN2. Thus, probably both mechanisms converge and conduce to thymocyte depletion but with different kinetics.

Notably, all DN thymocyte populations returned to the basal levels at 35 d.p.i., likely indicating a transition to the chronic phase of infection, in which the thymus environment is restored.

Surprisingly, we observed an increase in total thymic cell numbers, DP and SP, but not in DN thymocytes, at 7 d.p.i., suggesting that thymopoiesis was somehow stimulated at the beginning of the infection, an issue that deserves further investigation in the future. However, DP cell numbers subsequently decreased in agreement with previous observations [17]. Interestingly, this drop took place later than DN reduction.The decrease in DN and DP precursors is likely affecting CD4^+^ and CD8^+^ numbers that could compromise the T cell immune response [6]. Another remarkable finding is that the decrease in both CD4^+^ and CD8^+^ SPs thymocyte numbers was observed at 21 and 35 days post-infection, much later than the decrease in DP (13 d.p.i.). The reason for this is unclear. It may indicate that CD4^+^ and CD8^+^ SPs remain healthy in the thymus for at least 7 days without dying by apoptosis and/or being exported to the periphery as a consequence of its normal development. In this regard, the average life-span of SP in the thymus, is around 12 days for both CD4^+^ and CD8^+^ subpopulations [25]. Together, this suggests a minimal direct effect of infection on SP, being the observed effects likely secondary to DN-DP dynamics and their own life span.

Here, we found a significant increase in *Il6* mRNA as previously observed in heart tissue and its protein in plasma of infected mice [17]. In addition, there was a significant increase of *Il6ra* mRNA, but not *Il6st* mRNA, above to the basal levels. However, basal levels of expression of *Il6*, *Il6st* and *Il6ra* in the thymus were high as evidenced by ∆CT values around 19, 11 and 10, respectively. These lead us to hypothesize that IL-6 is triggering inflammation in the thymus, affecting differentiation from DN1 to DN2 stage.

In agreement with this hypothesis, we found that IL-6^−/−^ andIL-6^+/+^ infected mice presented similar depletion of DN1 stage cell numbers compared to uninfected mice, indicating that this does not depend on IL-6. Notably, there were higher numbers of DN2 and DN3 stages in IL-6^−/−^ infected mice compared to IL-6^+/+^ mice. Thus, the inhibition of the DN1 to DN2 transition caused by *T. cruzi* infection is likely to be mediated by IL-6.

It has been recently described that Sphingosine-1-phosphate receptor 1 (S1P1R)-mediates DN premature exit from the thymus since it was completely abrogated in S1P1R deficient mice [15]. Thus, the partial restoration of thymocyte stages observed in our IL-6^−/−^ infected mice model suggests that premature DN exit from the thymus cannot be completely attributed to IL-6.

As mentioned, many mechanisms were described to explain the thymic atrophy observed during *T. cruzi* infection. The significant increment in corticosterone levels we found is in agreement with the alterations on the hypothalamus-pituitary-adrenal (HPA) axis and endocrine homeostasis described during *T. cruzi* infection [26], that cause apoptosis of DP thymocytes. Moreover, blockade of glucocorticoid receptor activity prevented thymocyte apoptosis [11]. However, it occurred later than the bone marrow lymphopoietic defect and the alteration in DN differentiation.

Thus, the mechanism of *T. cruzi* thymic involution and dysfunction is likely to be multifactorial. Based on these results, we propose a dynamic model in which there is first an increase in the thymopoiesis, being the reason for that unknown. Later, a decrease in CLPs generation in the bone marrow may have a negative impact in all thymocyte stages. In addition, excessive IL-6 production, systemically and locally in the thymus may affect DN1 to DN2 transition. Finally, the additive effects on bone marrow precursor deficiency and IL-6 effect on DN1 to DN2 transition, combined with glucocorticoid-dependent thymocyte apoptosis and other mechanisms of thymocyte depletion, as premature DP and/or DN exit, would cause the severe drop on DP thymocytes. In summary, our results add further levels of complexity that will help to better understand thymic atrophy not only in *T. cruzi* infection. Finally, similar defects in DN differentiation may contribute to thymic atrophy in other infections where IL-6 is elevated.

## MATERIALS AND METHODS

### Parasites and mice

Female BALB/c mice (6 to 8-week-old) were purchased from Harlan-InterfaunaIberica and Charles River Laboratories España, and maintained at the Centro de Biología Molecular Severo Ochoa (CBMSO, CSIC-UAM, Madrid, Spain) animal facility. IL-6-knockout (IL-6^−/−^; B6.129S2-Il6 < tm1Kopf > /J) and C57BL/6J wild type mice were obtained from the Jackson Laboratory, Bar Harbor, ME, USA, and were maintained at the Animal Resource Facility of the CIBICI-CONICET (Argentina).

*In vivo* infections were performed with Y *T. cruzi* strain as described [27, 28]. Mice were infected with 2,000 blood trypomastigotes per mice by intraperitoneal injection. For BALB/c mice at 0, 7, 10 and 13 d.p.i., 3 mice were infected per experimental group. However, at 16, 21 and 35 d.p.i. the number of infected mice per experimental group was increased up to 15 in order to get at least 3 surviving mice in each experimental group that were analyzed. For IL-6^+/+^ and IL-6^−/−^ mice at 0 and 18 d.p.i. 3 mice were infected per group. All experiments were performed two times. Survival was monitored daily and parasitemia levels were checked every 2-3 days. Mice blood and tissues were collected at the indicated days post-infection (d.p.i.).

### Ethics statement

This study was carried out in strict accordance with the European Commission legislation for the protection of animals used for scientific purposes (Directives 86/609/EEC and 2010/63/EU). Mice were maintained under pathogen-free conditions at the CBMSO (CSIC-UAM) animal facility. The protocol for the treatment of the animals was approved by the ‘‘Comité de Ética de Investigación de la Universidad Autónoma de Madrid’’, Spain (permits CEI-14-283 and CEI-47-899). Experiments performed in Argentina followed the recommendations from the Guide for the Care and Use of Experimental Animals (Canadian Council on Animal Care) and approved by the National University of Cordoba and CIBICI-CONICET, Argentina. Animals had unlimited access to food and water. They were euthanized in a CO_2_ chamber and all efforts were made to minimize their suffering.

### Flow cytometry analysis

Flow cytometry was performed as previously described [28]. Briefly, FcγRs were blocked with anti CD16/CD32 antibody (Fc block) prior to staining with antibodies coupled to fluorophores. The flow cytometry staining antibodies used are listed in Supplementary Table 1. Bone marrow cells were isolated and stained with antibodies against Lineage markers (Lin: anti-CD4, anti-CD8, anti-B220, anti-Ter119, anti-CD11b, anti-Gr-1 and anti-DX5), Then lineage negative (Lin^neg^) gated cells were analyzed using Sca-1 and c-Kit markers, and CLPs identified as Lin^neg^ Sca1^neg^ c-Kit^med^. Thymocytes were isolated and stained with anti-CD4-CD8-CD25-CD44 antibodies. Analysis of DN, DP and SP stages was performed based on CD4 and CD8 staining, and DN1 to DN4 stages based on CD25 and CD44 staining on the Lin^neg^ gated population. Samples were analyzed in a FACS Canto II Cytometer (Becton Dickinson) using the FlowJo software (Tree Star, Inc. Oregon Corporation). The compensation controls of the labeled antibodies are shown in Supplemental Figure 2.

### mRNA analysis by quantitative reverse transcription PCR

For RNA extraction, thymuses were elicited and thymocytes suspensions obtained after mechanical disruption utilizing 40-μm mesh cell strainers (Falcon) and bone marrow cells were isolated from mice femurs. mRNAs were isolated utilizing TRIzol reagent (Invitrogen) as indicated by the manufacturer. Gene expression of IL-6 (*Il6*) or its receptor was analyzed by quantitative reverse transcription PCR (RTqPCR). Reverse transcription of total RNA was performed using the components of the High Capacity cDNA Reverse Transcription Kit (Applied Biosystems). Amplifications were performed using oligonucleotide probes (Supplementary Table 2) and the GoTaq qPCR MAster Mix (Promega) on an ABI PRISM 7900 HT instrument (Applied Biosystems). All samples were assayed in triplicate. Quantification of gene expression by real-time PCR was calculated by the comparative threshold cycle (CT) method as described in [29] (RQ = 2^-ΔΔCT^). All quantifications were normalized to the ribosomal *18S* control to account for the variability in the initial concentration of RNA and in the conversion efficiency of the reverse transcription reaction.

### TUNEL assay

Thymocytes were isolated from BALB/c mice at different days post-infection and processed with the *In situ* cell detection kit, TMR red (Roche) following the directions of the manufacturer and analyzed in a FACS Canto II Cytometer (Becton Dickinson) using the FlowJo software (Tree Star, Inc. Oregon Corporation).

### Corticosterone relative levels in plasma

Corticosterone relative levels were determined in mouse plasma by Metabolon Inc. using gas chromatography (GC)/mass spectrometry (MS) and liquid chromatography (LC)/MS platforms, raw data was normalized and results expressed as ScaledImpData as previously described [30].

### Statistical analysis

Data is shown as mean ± SEM. Statistical significance was evaluated by Student's *t*-test when two groups were compared, and one way ANOVA, followed by Tukey post-test, when different times were evaluated in an experimental group, using the GraphPad Prism 5.00 software (La Jolla, CA, USA).

## SUPPLEMENTARY MATERIALS FIGURES AND TABLE


