# Supplementary material for: Altered bone marrow lymphopoiesis and interleukin-6-dependent inhibition of thymocyte differentiation contribute to thymic atrophy during Trypanosoma cruzi infection
Source: Oncotarget. 2017 Jan 28;8(11):17551–61. doi: 10.18632/oncotarget.14886 (PMC5392268; doi:10.18632/oncotarget.14886)
Supplement: Supplementary file 1 [file oncotarget-08-17551-s001.pdf]

Altered bone marrow lymphopoiesis and interleukin-6-dependent inhibition of thymocyte differentiation contribute to thymic atrophy during *Trypanosoma cruzi infection*

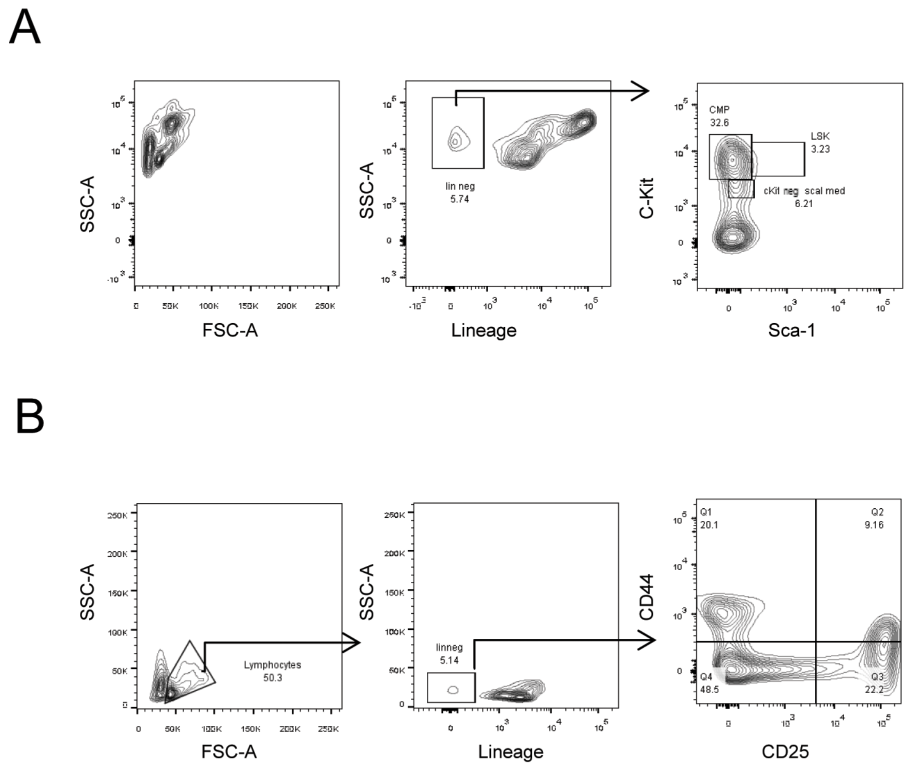

Figure S1: Gating strategy followed for flow cytometry analysis. Bone marrow cells and thymocytes were gated from SSC-A/FSC-A dot plots and showed two main populations. The thymocyte gate was drawn to exclude erythrocytes evidenced by Ter119 and CD71 staining (data not shown). (A) Hematopoietic precursor singlets from non-infected mice were gated from FSC-A/FSC-H to exclude cell doublets. Singlets were analyzed in Lineage/SSC-A dot plots and Lin^{neg} cells were gated. Then CLPs were identified in $Sca\text{-}1^{neg}$ $c\text{-}Kit^{med}$ gate. (B) Gating of the $Lineage^{neg}$ thymocytes allowed us to analyze CD25 and CD44 dot plots and identify DN subpopulations (DN1, DN2, DN3 and DN4).

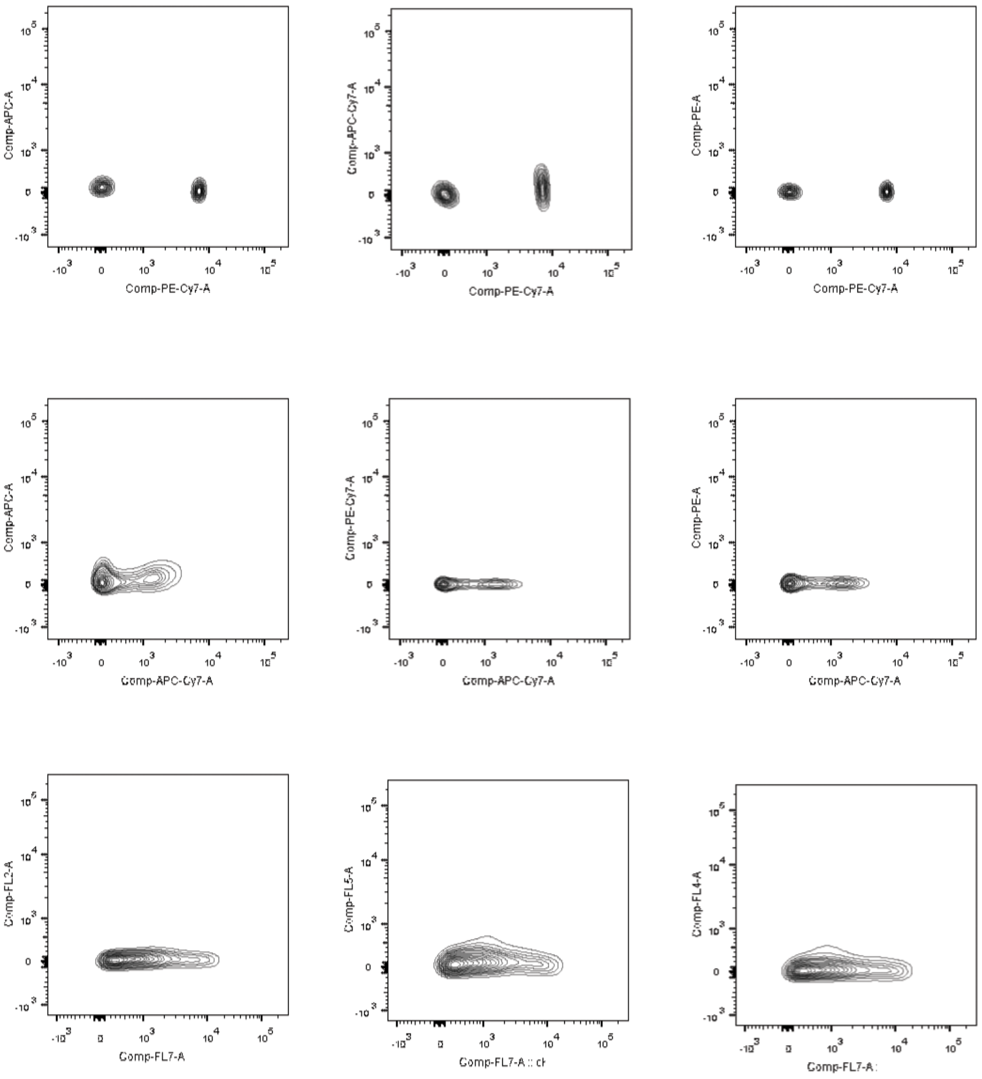

Figure S2: Compensation controls for flow cytometry analysis. Compensation controls using lymphocytes from non-infected mice and labeled antibodies.

S1 Table: Antibodies for flow cytometry. List of antibodies used in flow cytometry analysis including the target molecule, hybridoma clone, reference from provider and working dilution.

Antibody	Clone	Reference	Provider	Dilution
anti CD4 FITC	H129.19	553651	BD	1:500
anti CD4 PE	(L3T4)	553049	BD	1:500
anti CD8 APC	53-6,7	551162	BD	1:500
anti CD8 PE	53-6,7	553032	BD	1:500
anti CD25 APC	PC61,5	17-0251-82	eBioscience	1:500
anti CD44 biotin	IM7	553132	BD	1:500
Streptavidin APC-Cy7		554063	BD	1:500
anti Ter119 PE	TER-119	12-5921-81	eBioscience	1:500
anti cKit BV	2B8	562609	eBioscience	1:500
anti Sca-1 PECy7	D7	25-5981-81	eBioscience	1:500
anti B220 PE	RA3-6B2	553089	BD	1:500
anti CD11b PE	M1/70	557397	BD	1:500
anti Gr1 PE	RB6-8C5	12-5931-81	eBioscience	1:500
anti CD49B PE	DX5	553858	BD	1:500

S2 Table: Oligonucelotides for RTqPCR. Forward and reverse oligonucleotide sequences used for *Il6, Il6ra* and *Il6st* gene expression analysis by RTqPCR.

Gene	Oligonucleotide sequence forward	Oligonucleotide sequence reverse
Il6	5'-accagaggaaattttcaataggc-3'	5'-tgatgcacttgcagaaaaca-3'
Il6ra	5'-ggtgatcattcagggagcat-3'	5'-ggctcacaaaacagagaatgg-3'
Il6st	5'-tcatgttccttctatcgggtc-3'	5'-ctgagggaccggtggtgt-3'
